# Exercise reduces circulating biomarkers of cellular senescence in humans

**DOI:** 10.1111/acel.13415

**Published:** 2021-06-08

**Authors:** Davis A. Englund, Ayumi E. Sakamoto, Chad M. Fritsche, Amanda A. Heeren, Xu Zhang, Brian R. Kotajarvi, Denise R. Lecy, Matthew J. Yousefzadeh, Marissa J. Schafer, Thomas A. White, Elizabeth J. Atkinson, Nathan K. LeBrasseur

**Affiliations:** ^1^ Robert and Arlene Kogod Center on Aging Mayo Clinic Rochester MN USA; ^2^ Department of Physical Medicine and Rehabilitation Mayo Clinic Rochester MN USA; ^3^ Dan Abraham Healthy Living Center Mayo Clinic Rochester MN USA; ^4^ Center for Clinical and Translational Sciences Mayo Clinic Rochester MN USA; ^5^ Department of Biochemistry, Molecular Biology, and Biophysics University of Minnesota Minneapolis MN USA; ^6^ Department of Physiology and Biomedical Engineering Mayo Clinic Rochester MN USA; ^7^ Division of Clinical Trials and Biostatistics Department of Quantitative Health Sciences Mayo Clinic Rochester MN USA

**Keywords:** aging, immune cells, inflammation, senotherapeutics

## Abstract

Cellular senescence has emerged as a significant and potentially tractable mechanism of aging and multiple aging‐related conditions. Biomarkers of senescent cell burden, including molecular signals in circulating immune cells and the abundance of circulating senescence‐related proteins, have been associated with chronological age and clinical parameters of biological age in humans. The extent to which senescence biomarkers are affected by interventions that enhance health and function has not yet been examined. Here, we report that a 12‐week structured exercise program drives significant improvements in several performance‐based and self‐reported measures of physical function in older adults. Impressively, the expression of key markers of the senescence program, including *p16,* *p21, cGAS*, and *TNFα*, were significantly lowered in CD3^+^ T cells in response to the intervention, as were the circulating concentrations of multiple senescence‐related proteins. Moreover, partial least squares discriminant analysis showed levels of senescence‐related proteins at baseline were predictive of changes in physical function in response to the exercise intervention. Our study provides first‐in‐human evidence that biomarkers of senescent cell burden are significantly lowered by a structured exercise program and predictive of the adaptive response to exercise.

## Graphical abstract

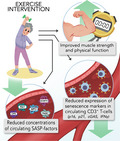

## 1 INTRODUCTION, RESULTS, AND DISCUSSION

Cellular senescence is a plausible and potentially tractable mechanism of aging. Molecular hallmarks of senescent cells, including the expression of cyclin‐dependent kinase inhibitors (CDKIs), DNA damage‐response proteins, and mediators and components of the senescence‐associated secretory phenotype (SASP), increase in multiple tissues with advancing age and chronic disease (Tuttle et al., 2020). Indeed, in mice, targeted elimination of senescent cells by genetic approaches and candidate senotherapeutic drugs restores tissue health and attenuates the progression of numerous age‐related conditions (Baker et al., 2016; Childs et al., 2016; Farr et al., 2017; Jeon et al., 2017; Roos et al., 2016; Schafer et al., 2017; Xu et al., 2015; Zhu et al., 2015).

The preclinical promise of senescent cell clearance as a translatable intervention for human health has highlighted the need for accessible and informative biomarkers of senescent cell burden. Two promising candidates, based on their cross‐sectional associations with chronological age and clinical indices of biological age (e.g., disease burden and frailty) in humans, include the expression of the CDKI *p16* in peripheral blood T cells and the circulating abundance of SASP proteins (Liu et al., 2009; Schafer et al., 2020). The responsiveness of these biomarkers of cellular senescence in older adults to intervention, however, has not yet been examined.

**FIGURE 1:**
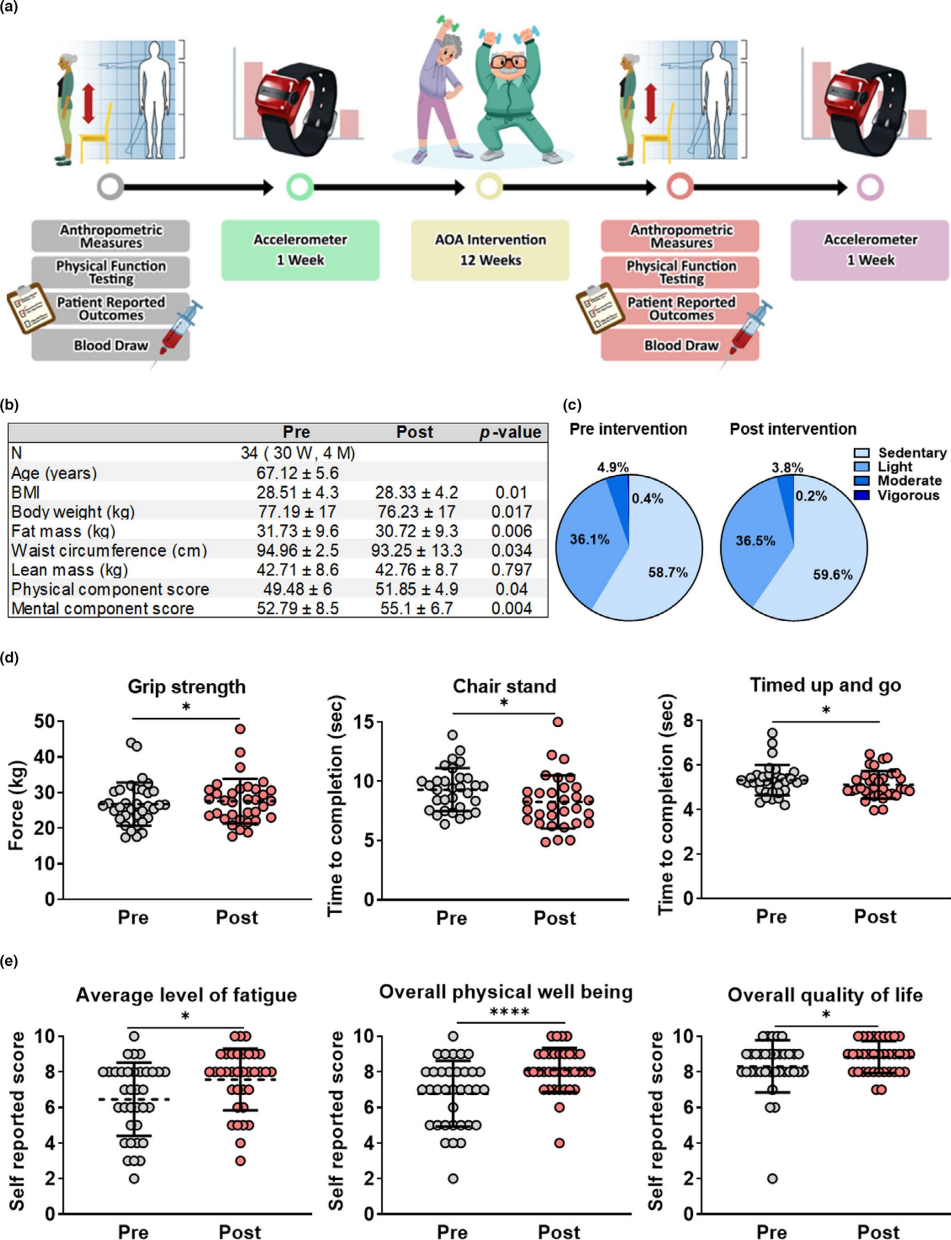
Twelve weeks of structured exercise improves body composition and physical function in older adults. (a) Study design schematic. (b) Subject characteristics at baseline and follow‐up; measures of body composition were assessed by DEXA scan and self‐reported physical and mental function was captured with the MOS SF‐12. (c) Levels of habitual physical activity before and after the intervention period, as assessed by accelerometry. (d) Clinical measures of physical function and strength before and after the intervention. (e) Self‐reported measures of functional status at baseline and follow‐up, as assessed by a linear analog self‐assessment scale. Results are mean ± SD. *p < 0.05, ****p < 0.0001

Exercise remains the most promising intervention to improve physical function in older adults, and prior observations in preclinical models and humans suggest it may influence senescent cell burden (Chen et al., 2021). Correspondingly, we studied the effects of a 12‐week structured exercise intervention comprised of progressive strength and endurance training 2 days/weeks on the molecular phenotype of isolated CD3^+^ T cells and the circulating concentrations of SASP proteins in older adults (Figure 1a). We hypothesized that structured exercise would drive significant reductions in biomarkers of cellular senescence.

Following the intervention, participants exhibited significant improvements in body composition, evidenced by significant reductions in body weight, BMI, waist circumference, and fat mass (Figure 1b), despite no change in habitual physical activity levels (Figure 1c). Measures of muscle performance and physical function also improved, including grip strength, repeated sit‐to‐stand time, and the timed up and go (TUG) test (Figure 1d). While we did not have a control group for direct comparison, improvements in objective assessments were paralleled by improvements in patient‐reported outcomes, including fatigue, physical well‐being, and overall quality of life, increasing our confidence in these findings (Figure 1e).

**FIGURE 2:**
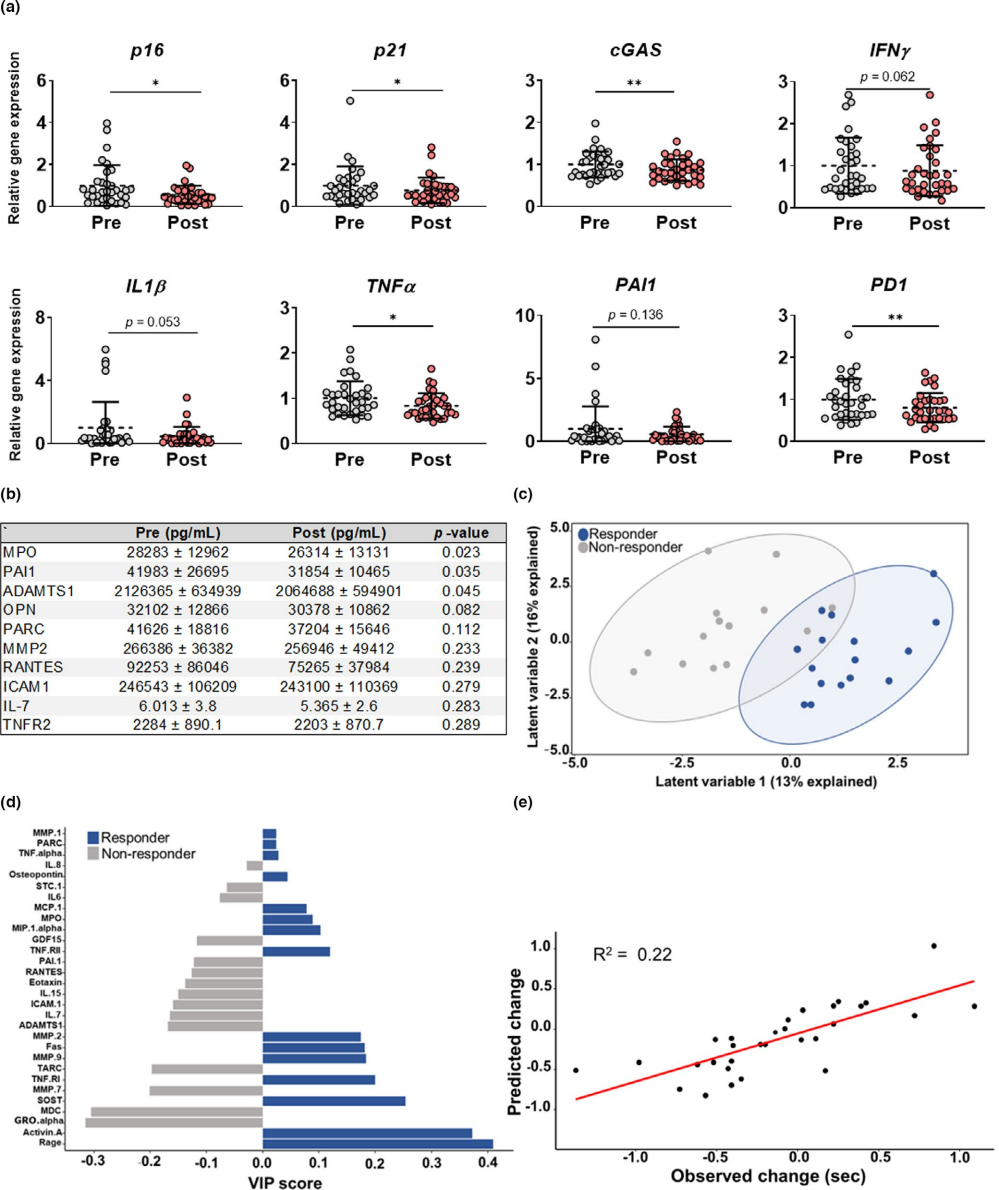
Biomarkers of cellular senescence are predictive of functional adaptation to exercise. (a) Expression of cellular senescence markers in CD3^+^ T cells at baseline and following the intervention period. (b) Concentrations of circulating senescence‐related proteins before and after the intervention. (c) The PLSDA‐derived index of senescent cell burden differentiates between subjects who showed improvements in the timed up and go measure (responders [blue]) and those who did not (non‐responders [gray]) in response to the intervention. (d) VIP scores, which rank SASP factors on their importance for differentiating between responders (blue) and non‐responders (gray). (e) Predicted and observed change in gait speed using the SASP index. Results are mean ± SD. *p < 0.05, **p < 0.01

To assess the impact of exercise on biomarkers of cellular senescence, we first profiled peripheral blood CD3^+^ T cells by qPCR before and after the intervention. Impressively, exercise significantly and consistently reduced the expression of the CDKIs *p16* and *p21* as well as mediators and constituents of the SASP. Components of the cGAS‐STING pathway (e.g., *cGAS,* *IFNγ*, and *TNFα*), which triggers inflammation and reinforces the senescence program (Li & Chen, 2018), were also reduced (Figure 2a). Next, we examined plasma concentrations of prototypical SASP components, including cytokines, chemokines, matrix remodeling proteins, and growth factors, using a multiplex assay (Schafer et al., 2020). The intervention modestly but again consistently lowered the circulating abundance of multiple senescence‐related proteins, including the inflammatory factor, myeloperoxidase (MPO), and thrombotic, fibrinolytic, and inflammatory factor, serpin E1 (PAI1) (Figure 2b). Based on these findings and the diversity of our SASP panel, we utilized partial least squares discriminant analysis (PLSDA) to integrate values for baseline protein concentrations and calculate a multi‐dimensional SASP index for each participant.

Notably, the SASP index differentiated participants who responded favorably to the intervention (responders) from those that did not (non‐responders), based on the TUG measure, an integrative measure of physical function (Figure 2c). Variable importance projection (VIP) calculations, which rank individual SASP factors based on their overall importance for separating responder and non‐responder data clouds, showed that SASP factors had a range of importance for distinguishing responders from non‐responders (Figure 2d). Moreover, the SASP index was predictive of the degree to which participants' performance changed in response to the exercise intervention (Figure 2e).

Our data demonstrate the responsiveness of the molecular phenotype of CD3^+^ T cells and components of the SASP to intervention, which provides a critical level of support for their use as biomarkers of senescence. This criterion adds to prior work that has established other key attributes of these candidates, including relevance to aging, measurement feasibility and reliability, and associations with clinical outcomes (Justice et al., 2018; Liu et al., 2009; Schafer et al., 2020).

Biomarkers offer significant utility to clinical trials as they can help determine indications and even individuals that may be most responsive to intervention. Further, our data suggest senescence transcripts in CD3^+^ T cells and a diverse panel of SASP proteins in plasma can potentially serve as surrogate endpoints that, in combination with predictive analytics like PLSDA, forecast clinical outcomes. This is of particular value given the time course for meaningful changes in clinical endpoints, such as physical, cardiovascular, and cognitive function measures, disease progression, and, of course, mortality (Biomarkers Definitions Working & G, 2001). As core properties of the senescence program, alterations in CDKI expression and SASP abundance may also reflect target engagement and the bioactivity of emerging interventions. Additional research is needed to understand the extent to which these circulating biomarkers reflect senescent cell burden in specific tissues and respond to senotherapeutic interventions in the context of age‐related diseases and geriatric syndromes.

Our study highlights the impact of exercise on the fundamental biology of aging and, in turn, human health. Twelve weeks of structured exercise did not alter habitual physical activity, a finding that increases our confidence that the changes in senescence biomarkers can be ascribed to the exercise program itself, and not extrinsic alterations in participant behavior. We do note that including a control group in the study design would have further reinforced these findings. Additional research and advocacy for lifestyle interventions as a means to counter aging and extend human healthspan is warranted.

## CONFLICT OF INTEREST

The authors declare no conflicts of interest. ClinicalTrials.gov Identifier: NCT04897373.

## AUTHOR CONTRIBUTIONS

NL provided the study design. MY involved in the study design. CF, BK, DL, and MS involved in subject recruitment and human data collection. DE, AS, AH, TW, EA and NL acquired, analyzed, or interpreted the data. DE and AS drafted the manuscript. All authors reviewed the manuscript.

## Supporting information

Supplementary Material

## Data Availability

The data that support the findings of this study are available from the corresponding author upon reasonable request.
