# Supplementary material for: Exercise reduces circulating biomarkers of cellular senescence in humans
Source: Aging Cell. 2021 Jun 8;20(7):e13415. doi: 10.1111/acel.13415 (PMC8282238; doi:10.1111/acel.13415)
Supplement: Supplementary file 1 — Supplementary Material [file ACEL-20-e13415-s001.docx]

**METHODS**

Older adults were recruited from the Dan Abraham Healthy Living Center

(DAHLC) at Mayo Clinic. The DAHLC’s mission is to improve the health and well-being of Mayo Clinic employees, retirees, volunteers & their spouses by implementing effective wellness, practice, education and research strategies. Of the approximately 16,000 members, more than 4,000 are over the age of 60 years. To be included in the trial, participants had to provide informed consent and be able to get down and off the floor with little assistance. ClinicalTrials.gov Identifier: NCT04897373

**Intervention**

All enrolled participants underwent an exercise intervention that involved two structured group sessions per week for twelve weeks. Each session was approximately 90 minutes in duration and consisted of a brief warm-up, balance exercises, resistance training, cardiovascular exercise, and a cool-down period. Progression throughout the twelve weeks was individualized to the participant. The aim of the intervention was to improve balance, mobility, whole-body strength, and cardiovascular health. Overall, subject adherence was very high (mean [standard deviation] attendance was 89.65% [7.94%]) with a range of 75% - 100% attendance.

**Accelerometry**

Participants wore a belt-based ActiGraph tri-axial accelerometer at the waist during waking hours (any time except showering and sleeping) over a course of seven days. The accelerometer was used before and after the intervention to assess the magnitude and frequency of everyday physical activity (Freedson et al., 1998). Accelerometer data was sampled at a rate of 30 hz and summarized using 1 minute epochs. Data was analyzed only if the subject wore the monitor for a minimum of 10 hours each day and for a minimum of 4 days. ActiLife software was used to calculate activity counts, sedentary bouts, and step counts for each subject.

**Body composition**

Dual Energy X Ray Absorptiometry (DEXA (Lunar iDXA; GE Medical Systems, Chicago, Illinois)) total body scans were utilized at baseline and post-intervention to measure total body fat mass and lean mass in participants.

**Waist Circumference**

Waist circumference was obtained with the participant’s abdomen free of all clothing and accessories. The participant was positioned with feet shoulder width apart and arms crossed over the chest in a relaxed manner. The waist circumference measurement was taken at the top of the iliac crest by positioning the tape directly around the abdomen so that the inferior edge of the tape is at the level of the landmarked point. At the end of the normal expiration, the measurement was taken to the nearest 0.1cm.

**Muscle strength and function**

*Grip Strength*

Grip strength was measured using a *Jamar* handgrip dynamometer. For this test, the participant was seated and the tested arm was flexed at 90 degrees. The subjects gripped the apparatus with maximal force for as long as possible when verbally instructed to do so.

*Chair Stand*

A Chair Stand test was used to quantify lower body strength. Before performing five consecutive trials, each subject performed a single chair stand to confirm that they were capable of the motion. Each chair stand was timed and scored.

*Timed up and Go*

The participants were instructed to stand up from the seated position and to walk at a normal and comfortable pace for three meters before turning around to return to the chair. The get up and go test is used as an assessment of overall gait and mobility (Steffen et al., 2002).

**Patient reported outcomes**

*SF-12 Survey*

The SF-12 survey is comprised of twelve questions that examine the influence of an individual’s health on their everyday activities. The results of the SF-12 survey yield a Physical Component Score (PCS) and Mental Component Score (MCS) which can be used as a comprehensive assessment of overall quality of life (Younsi, 2015).

*Linear Analogue Self-Assessment Scale*

The linear analogue self-assessment consisted of ten core questions that addressed physical, emotional, mental, spiritual, and social wellbeing (Locke et al., 2007). The participants answered each of the questions based on a scale from 0 (as bad as can be) to 10 (as good as can be).

**Blood sample preparation**

Blood draws were conducted at baseline and after the intervention. 50 mL of blood was collected from each participant. Immediately after collection, a portion of the blood sample was used to prepare plasma for further downstream analyses to examine concentrations of various proteins (see below for more details). Another portion (8ml) was used for CD3^+^ cell isolation for further downstream analyses to determine expression of various mRNAs (see below for more details).

**Circulating senescence factors**

The concentration of various proteins of interest in patient plasma samples (500 ul) were quantified using commercially available multiplex magnetic bead immunoassays (R&D Systems) based on Luminex xMAP multianalyte profiling platform and analyzed on a MAGPIX System (Merck Millipore). All assays were performed according to the manufacturer’s protocols (Schafer et al., 2020).

**CD3^+^ T cell isolation and characterization**

CD3^+^ T cells were isolated from whole blood samples using automatic magnetic-activated cell sorting technology. The cells were first tagged with CD3^+^ Microbeads (Miltenyi Biotec) before being processed by the AutoMACS^TM^ pro separator (Miltenyi Biotec). The isolated cells were then spun down and stored in Trizol (Invitrogen), and RNA isolation was completed per the manufacturer’s instructions. RNA concentrations were assessed by NanoDrop (Thermo Fisher Scientific). cDNA was synthesized using M-MLV reverse transcriptase (Invitrogen), and qPCR was performed with PerfeCTa FastMix II (QuantaBio) and the Applied Biosystems StepOne Plus Real-Time PCR system. Gene expression was analyzed by ∆∆CT method and normalized to the reference gene, TATA-Box Binding Protein (TBP). RT–PCR primers used in this study are listed in Supplementary Table 1 (Integrated DNA Technologies, Coralville, IA).

**Statistics**

Results are presented as mean ± standard deviation of the mean (SD) and compared using a paired two-tailed Student’s t-test. Partial least squares discriminant analysis (PLSDA) was used to calculate the linear combination of the biomarkers that best discriminates between responders and non-responders. Similarly, partial least squared regression (PLSR) was fit using the change in the TUG measure as the endpoint. Prior to the PLS analyses, biomarkers were standardized by applying a log transformation as needed, then subtracting the mean and dividing by the standard deviation so that all the biomarkers where symmetrical and on a similar scale. Variable importance (VIP) was estimated using the absolute value of the loadings for the first component from the PLSDA analysis, then multiplying by the square-root of the number of biomarkers to put on a nice scale. Essentially, because the biomarkers on are on the same scale, it provides a measure of the relative weighting of each variable in differentiating between the two groups. Data were analyzed using the GraphPad Prism and R; in R, the mixOmics package was used for the PLS analysis.

**Supplementary Table 1**

| **Gene** | **Primer 1** | **Primer 2** | **Probe** |
| --- | --- | --- | --- |
| CDKN2A | CCAACGCACCGAATAGTTACG | GCGCTGCCCATCATCATG | CCTGGATCGGCCTCCGAC |
| CDKN1A | GAGACTAAGGCAGAAGATGTAGAG | GCAGACCAGCATGACAGAT | TTCCTCTTGGAGAAGATCAGCCGG |
| TNFα | TCAGCCTCTTCTCCTTCCT | TTCGAGAAGATGATCTGACTGC | CCGATCACTCCAAAGTGCAGCAG |
| IFNγ | GCAACAAAAAGAAACGAGATGAC | CGACAGTTCAGCCATCACTT | TCGGTAACTGACTTGAATGTCCAACGC |
| IL1β | GAACAAGTCATCCTCATTGCC | CAGCCAATCTTCATTGCTCAAG | AGAAGTACCTGAGCTCGCCAGTGA |
| PAI1 | TGACAACAGGAGGAGAAACC | GAGCTCCTTGTACAGATGCC | TGCCCTTGTCATCAATCTTGAATCCCA |
| cGAS | GAGCTACTATGAGCACGTGAAG | AGTAATATGCACGAGTGTTGGA | TCTGCACCTAATGAATTTGATGTCATGTTTAAACTG |
| PD1 | CTGGTGCTGCTAGTCTGG | CCATAGTCCACAGAGAACACAG | CCTGGCTCCTATTGTCCCTCGTG |

**Supplementary References**

Freedson, P. S., Melanson, E., & Sirard, J. (1998). Calibration of the computer science and applications, inc. accelerometer. *Medicine and science in sports and exercise*, *30*(5), 777-781.

Locke, D. E., Decker, P. A., Sloan, J. A., Brown, P. D., Malec, J. F., Clark, M. M., Rummans, T. A., Ballman, K. V., Schaefer, P. L., & Buckner, J. C. (2007). Validation of single-item linear analog scale assessment of quality of life in neuro-oncology patients. *Journal of pain and symptom management*, *34*(6), 628-638.

Schafer, M. J., Zhang, X., Kumar, A., Atkinson, E. J., Zhu, Y., Jachim, S., Mazula, D. L., Brown, A. K., Berning, M., Aversa, Z., Kotajarvi, B., Bruce, C. J., Greason, K. L., Suri, R. M., Tracy, R. P., Cummings, S. R., White, T. A., & LeBrasseur, N. K. (2020). The senescence-associated secretome as an indicator of age and medical risk. *JCI Insight*, *5*(12). <https://doi.org/10.1172/jci.insight.133668>

Steffen, T. M., Hacker, T. A., & Mollinger, L. (2002). Age- and gender-related test performance in community-dwelling elderly people: Six-Minute Walk Test, Berg Balance Scale, Timed Up & Go Test, and gait speeds. *Phys Ther*, *82*(2), 128-137. <https://doi.org/10.1093/ptj/82.2.128>

Younsi, M. (2015). Health-related quality-of-life measures: evidence from Tunisian population using the SF-12 health survey. *Value in health regional issues*, *7*, 54-66.
